# Supplementary material for: Camsap2a regulates actomyosin flow and Rab5ab-mediated macropinocytosis in the yolk cell during zebrafish epiboly
Source: Development. 2026 Feb 2;153(3):dev204843. doi: 10.1242/dev.204843 (PMC12912265; doi:10.1242/dev.204843)
Supplement: Supplementary information [file develop-153-204843-s1.pdf]

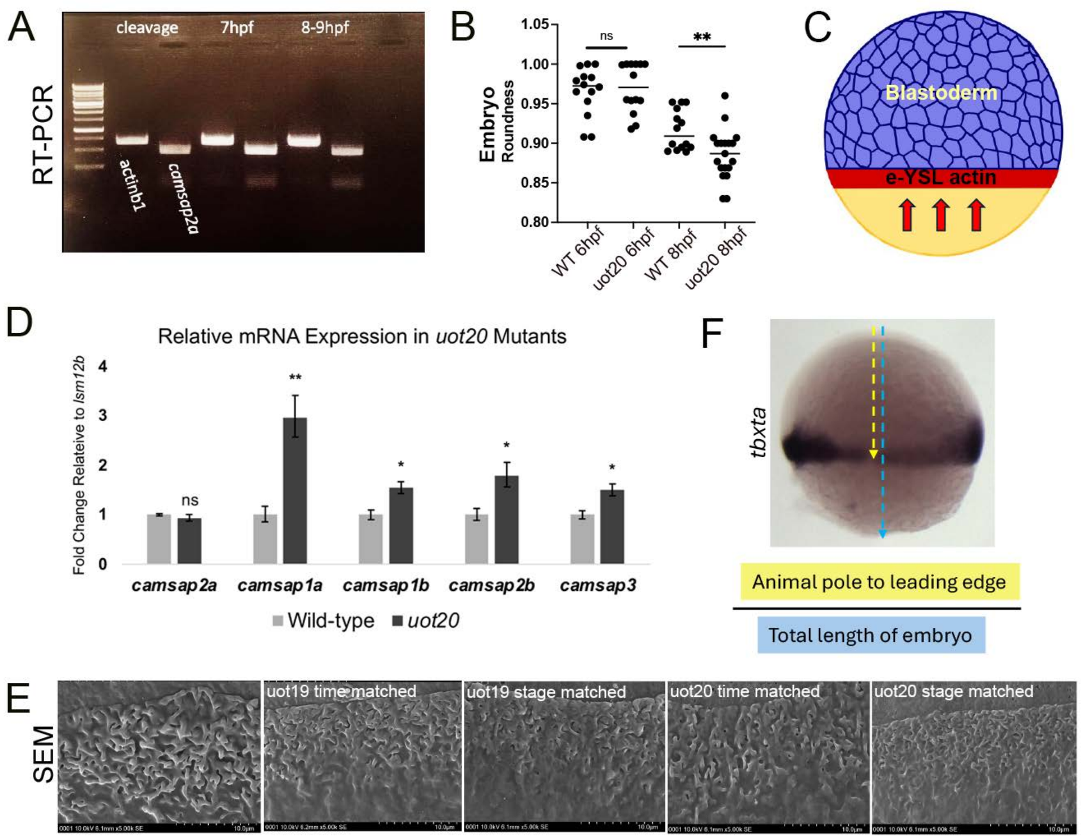

Fig. S1. Analysis of gene expression and morphology in *MZcamsap2a* mutant embryos

(A) PCR amplification of *actinbl* (positive control) and *camsap2a* from cDNA made from embryos at the indicated stages. First lane, 1 kb ladder; last lane, no reverse transcriptase control.

(B) Roundness quantification in wild type (n l4) and *MZcamsap2a (uot20,* n = l9) mutant embryos at 6hpf and 8hpf. Mean: SEM, Welch's t-test, ** p = 0.0043 <0.05.

(C) Schematic embryo showing blastoderm and e-YSL. The actomyosin ring accumulates by upward flow (arrows) and forms in the e-YSL region. To quantify yolk actin, myosin and macropinocytosis , analyses were limited to the e-YSL region. In some cases, e-YSL fluorescence was normalized to fluorescence of the whole blastoderm , see Methods for details. (D) Mean expression levels of *camsap* orthologs *(camsapla, camsaplb, camsap2a, camsap2b* and *camsap3)* relative to *lsm12b* expression in wild type and *MZcamsap2a* embryos *(uot20,* N = 3, 50 embryos/N). Statistical analysis was performed on normalized expression levels using two-tailed student's t-test with 95% confidence interval. Mean: SEM; *, p<0.05. **, p<0.01. (E) Scanning electron micrographs of e-YSL in wild type embryo at 75% epiboly (8hpf), and time-matched (TM) and stage-matched (SM) mutant embryos. Ruffled region is the e-YSL, embryos oriented animal pole up. (F) *tbxta* expression in a wild-type embryo illustrating how epiboly progression was quantified in Fig. 8A and Fig. 9E,F.

Development • Supplementary information

Table S1. Concentrations of injected plasmids, mRNA and protein

**Plasmids**	**Amount**
dUAS:H2B-RFP-EB3-GFP	70 pg
pzTol2[Exp]-{wnt8 promoter}>{camsap2a}	100 pg
FP2: Rab5ab	100 pg
FP2: CA-Rab5ab	95 pg
**mRNA**	
Gal4	84 pg
EMTB-3xGFP	70 pg
**Protein**	
Rhodamine-actin (protein)	1 µg

Table S2. Constructs, plasmids and protein sources

**Constructs**	**Source**
pCS2+	Gift from Richard Harland (Turner and Weintraub, 1994)
pzTol2[Exp]-{wnt8 promoter}>{camsap2a}	VectorBuilder Inc. (custom plasmid synthesis, Vector ID: VB241120-1300mzb)
pFP2	Gift from Arne Lekven (Narayanan and Lekven, 2012)
dUAS:H2B-RFP-EB3-GFP	Gift from Ulrike Theisen (Fei et al., 2019)
pCS2+-EMTB-3xGFP	Gift from William Bement (Addgene plasmid #26741; htttp://n2t.net/addgene:26741; RRID:Addgene_26741 (Miller & Bement, 2009)
pDONOR221	Thermo Fisher Scientific 12536017
pCSeGFPDest	Gift from Nathan Lawson (Addgene plasmid 13071; http://n2t.net/addgene;13071; RRID: Addgene_13071) (Villefranc et al., 2007)
Rhodamine-actin	Cytoskeleton, APHR-A

Development • Supplementary information
